# Reduced cognitive function during a heat wave among residents of non-air-conditioned buildings: An observational study of young adults in the summer of 2016

**DOI:** 10.1371/journal.pmed.1002605

**Published:** 2018-07-10

**Authors:** Jose Guillermo Cedeño Laurent, Augusta Williams, Youssef Oulhote, Antonella Zanobetti, Joseph G. Allen, John D. Spengler

**Affiliations:** Exposure, Epidemiology, and Risk Program, Department of Environmental Health, Harvard T.H. Chan School of Public Health, Boston, Massachusetts, United States of America; University of Wisconsin, Madison, UNITED STATES

## Abstract

**Background:**

In many regions globally, buildings designed for harnessing heat during the cold exacerbate thermal exposures during heat waves (HWs) by maintaining elevated indoor temperatures even when high ambient temperatures have subdued. While previous experimental studies have documented the effects of ambient temperatures on cognitive function, few have observed HW effects on indoor temperatures following subjects’ habitual conditions. The objective was to evaluate the differential impact of having air conditioning (AC) on cognitive function during a HW among residents of AC and non-AC buildings using a prospective observational cohort study.

**Methods:**

We followed 44 students (mean age = 20.2 years; SD = 1.8 years) from a university in the Greater Boston area, Massachusetts in the United States living in AC (*n =* 24) and non-AC (*n =* 20) buildings before, during, and after a HW. Two cognition tests were self-administered daily for a period of 12 days (July 9–July 20, 2016), the Stroop color-word test (STROOP) to assess selective attention/processing speed and a 2-digit, visual addition/subtraction test (ADD) to evaluate cognitive speed and working memory. The effect of the HW on cognitive function was evaluated using difference-in-differences (DiD) modelling.

**Findings:**

Mean indoor temperatures in the non-AC group (mean = 26.3°C; SD = 2.5°C; range = 19.6–30.4°C) were significantly higher (*p <* 0.001) than in the AC group (mean = 21.4°C; SD = 1.9°C; range = 17.5–25.0°C). DiD estimates show an increase in reaction time (STROOP = 13.4%, *p <* 0001; ADD = 13.3%, *p <* 0.001) and reduction in throughput (STROOP = −9.9%, *p <* 0.001; ADD = −6.3%, *p =* 0.08) during HWs among non-AC residents relative to AC residents at baseline. While ADD showed a linear relationship with indoor temperatures, STROOP was described by a U-shaped curve with linear effects below and above an optimum range (indoor temperature = 22°C–23°C), with an increase in reaction time of 16 ms/°C and 24 ms/°C for STROOP and ADD, respectively. Cognitive tests occurred right after waking, so the study is limited in that it cannot assess whether the observed effects extended during the rest of the day. Although the range of students’ ages also represents a limitation of the study, the consistent findings in this young, healthy population might indicate that greater portions of the population are susceptible to the effects of extreme heat.

**Conclusions:**

Cognitive function deficits resulting from indoor thermal conditions during HWs extend beyond vulnerable populations. Our findings highlight the importance of incorporating sustainable adaptation measures in buildings to preserve educational attainment, economic productivity, and safety in light of a changing climate.

## Abstract 2

Jose Guillermo Cedeño Laurent and colleagues document the detrimental effects of heatwaves on cognition among a group of students

## Author summary

**Why was this study done?:**

- Heat waves (HWs) have devastating consequences for public health globally.
- Buildings can exacerbate temperature exposures during HWs by maintaining high indoor temperatures overnight even when high ambient temperatures have subdued.
- Prior experimental studies have documented the effects of temperature on cognitive function, but no field studies have observed how indoor temperatures during HWs impact cognition, even though adults in the United States spend upwards of 90% of their time indoors.

**What did the researchers do and find?:**

- We evaluate the impact of having air conditioning (AC) during a HW on cognitive function among residents of AC and non-AC buildings using a prospective, observational cohort study.
- A cohort of university students was recruited from 2 campus residence types—AC (*n =* 24) and non-AC (*n =* 20)—and followed over 12 consecutive days in the summer of 2016.
- Students living in non-AC spaces experienced significant decrements on cognitive test performance. Results show an increase in reaction time (Stroop color-word test [STROOP] = 13.4%, *p <* 0001; 2-digit visual addition/subtraction test [ADD] = 13.3%, *p <* 0.001) and reduction in throughput (STROOP = −9.9%, *p <* 0.001; ADD = −6.3%, *p =* 0.08) during HWs among non-AC residents relative to AC residents at baseline.

**What do these findings mean?:**

- Future studies are needed to understand the duration of these effects to determine how the implications could extend to larger sectors of the population and could have significant impacts on educational attainment, economic productivity, and workplace safety.
- Given that existing adaptation solutions (e.g., AC) represent a positive feedback loop amplifying the effects of climate change, our findings highlight the need to provide sustainable adaptation solutions to foster adequate cognition during extreme heat events.

## Introduction

Heat waves (HWs) have devastating consequences for public health globally. Exposure to higher temperatures results in the human body’s inability to thermoregulate, leading to both indirect and direct health impacts, related to cardiovascular, respiratory, renal, cerebrovascular, and diabetes-related morbidity and mortality [1–6]. Estimates of heat-related mortality vary by location and population. In the United States, extreme heat exposure is the leading cause of death of all meteorological phenomena, responsible for over 7,000 deaths from 1999 to 2010 [7]. Previous studies have shown that heat-related mortality is punctuated by high-profile acute events like the HW in Europe in 2003 that claimed 70,000 lives and India in 2015 that was responsible for 2,300 heat-related deaths [8]. As global temperatures warm, temperatures that are currently thought of as extreme will become more common [9]. The changing climate has important heat-related public health implications. Across the globe, 2016 has been the warmest year in the past 200 years of recorded history [10], and a warmer climate in the future is expected to result in tens of thousands of excess deaths per year in the US by the year 2100 [11]. In addition to increasing overall mean temperature, climate change is projected to increase the frequency, duration, and intensity of HWs [12–15].

Historically, the public health impacts of HWs have been primarily conducted through epidemiologic studies using outdoor temperature records as the measure of exposure [16–18]. Adults in the US, however, spend about 90% of their time indoors [19], making past heat-related health research subject to exposure misclassification if the outdoor temperature values do not represent the indoor exposures. Evidence of the effects of extreme heat also stems from controlled, laboratory chambers with experimental temperature regimes [20–22]. The impacts of extreme heat are compounded in real life by behavioral factors that modify exposure [23–25] (e.g., sleep, hydration, physical activity, or air conditioning [AC]).

Furthermore, assessing extreme heat impacts has focused, primarily, on vulnerable populations like the very young or the elderly. Yet the health effects of extreme heat events can be experienced in the general population, resulting in subclinical symptoms, like cognitive function deficits [16,21,26]. A U-shaped response curve has been utilized to describe how exposures to extremely low or high temperatures result in decreased cognitive performance [16,21,27]. However, this has been based on findings from controlled, experimental settings or by utilizing outdoor temperature as the exposure metric. Therefore, field studies in real-world settings are needed to understand the relationship between extreme heat exposures in indoor environments, health, and cognition while controlling for important behavioral factors that affect exposure and risk.

Our prospective observational cohort study examined relationships between indoor environmental conditions, heat exposures, sleep, and cognitive function between young adults living in central AC and non-AC residence halls on a university campus before, during, and after a HW during the summer of 2016.

## Methods

### Study design

A cohort of university students was followed over 12 consecutive days (July 9–July 20, 2016) in the Greater Boston, Massachusetts in the US. The study started with a baseline period of 5 days of seasonable outdoor temperatures (mean = 20.4°C; range = 15.3–30.6°C) before the onset of a HW. We relied on the definition of HW used by the National Oceanic and Atmospheric Administration (NOAA), in which a HW consists of a period of 2 or more days of “abnormally high air temperature and humidity.” Our criteria for abnormally high temperature threshold was based on a daily maximum outdoor temperature (T_out,max_) of 32.2°C (90°F), which corresponds to the 91st percentile of average high temperatures in Boston during the June to September period for 10 years prior to the study. The onset of the study was based on meteorological forecasts indicating near-average summer temperatures for several days, followed by a period of extreme heat, acknowledging that the study phase durations would vary with the forecast. The observed weather conditions allowed for an uninterrupted 12-day study (July 9–July 20) comprising the following 2 periods: (1) an initial 5-day baseline period of seasonable temperature and (2) the HW period, consisting of 5 days of abnormally high temperatures (mean T_out,max_ = 33.4°C; range = 27.8–35.6°C) and a 2-day cooldown (mean T_out,max_ = 28.1°C; range = 27.8–28.3°C).

### Study participants

Students were assigned to their residences at the beginning of the summer and independently from their enrollment in this study. Students were recruited from 2 campus residence types: AC (*n =* 24) and naturally ventilated (non-AC, *n =* 20). AC study sites consisted of adjacent, 6-story buildings constructed in the early 1990s, with operable windows and central AC. The non-AC study sites consisted of low-rise, Neo-Georgian-style buildings with thick masonry walls constructed between 1930 and 1950 with an approximate 30% window-to-wall ratio. Prospective participants received details of the study at informative meetings held at each of the study sites. Recruitment occurred on a rolling basis until recruitment targets were met; the research team only required that groups from both building types were balanced in terms of student age and sex. Inclusion criteria required that the student was at least 18 years of age, had no history of alcohol or drug abuse, no history of or current pregnancy, and met a set of predetermined health conditions (was not using oral or intravenous antibiotics or chemotherapy, was not using prednisone or NSAIDs, did not currently have acute infectious disease [cold/flu, gastroenteritis, etc.], had not been previously self-diagnosed with a chronic inflammatory or autoimmune disease, was not taking any prescription sleep medications, and had no previously diagnosed sleep disorder). There were no significant differences in the prevalence of preexisting health conditions between students living in naturally and mechanically ventilated buildings (Table 1). Students chose their summer housing preferences on a first-come, first-serve basis in a manner that was not expected to be associated with exposure or outcome. Therefore, we assumed that these populations were exchangeable and that living in any one type of ventilation was independent of the student’s demographics, health status, or potential health outcomes. The Institutional Review Board (IRB) at the Harvard T.H. Chan School of Public Health approved this study.

**Table 1:** Baseline demographics of study participants: Students living in Greater Boston area, MA during summer 2016.

	No-AC (*n =* 20)	AC (*n =* 24)	*p*-value
Age (years)	20.3 ± 2.4 (18–29)	20.1 ± 1.1 (18–23)	0.72
Race, nonwhite (%)	14 (66.7)	13 (54.2)	0.39
US-born (%)	13 (61.9)	18 (75.0)	0.34
Male (%)	11 (52.4)	12 (50.0)	0.87
Excellent self-assessment of health	8 (38.1%)	6 (25.0%)	0.34
Very good to good self-assessment of health	13 (61.9%)	17 (70.8%)	0.53
No history of sleep medications (%)	19 (90.5)	24 (100)	0.12
Moderately to very physically active (%)	17 (80.0)	20 (83.4)	0.83
Smoke (%)	3 (15%)	0 (0%)	0.17
Use of diuretics	1 (5%)	0 (0%)	0.92

Abbreviation: AC, air conditioning.

### Survey instruments

#### Baseline survey

Consented students completed a baseline survey on demographics (e.g., age, gender, height, weight, smoking status, race, ethnicity, and use of contacts, hearing aids, and diuretics) and sleep quality during the week prior to the study. The survey included questions on perception of and satisfaction with indoor environmental quality (i.e., thermal comfort, indoor air quality, acoustics, and lighting) in the bedroom of their summer residence.

#### Daily survey and cognitive tests

Students were sent an electronic survey on their smartphones every morning and were instructed to complete it after waking up, while inside their bedroom. The survey started with a battery of 2 self-administered cognition tests: cognitive speed and inhibitory control were evaluated by the Stroop color-word test (STROOP), and cognitive speed and working memory were assessed by a 2-digit, visual addition/subtraction test (ADD). These tests have been used previously based on their sensitivity measuring the effects of hyperthermia on executive control reaction time and working memory [22,27–29]. The effects of hyperthermia on similar executive function and working memory tests have also been studied in combination with evaluations of activity and functional connectivity in the brain [26,30]. STROOP entailed 24 trials of congruent, incongruent, and neutral word-color stimuli, shown at the center of the student’s smartphone display. The student was expected to correctly identify the color of the displayed word and tap 1 of the 4 buttons (i.e., red, blue, green, or yellow) on the touch screen corresponding to the shown color. ADD had 10 mathematical addition and subtraction trials of 2-digit numbers, answered via the smartphone touch screen keyboard. Five performance metrics were obtained from the cognitive tests: cognitive speed on the STROOP incongruent trials and ADD were assessed by reaction time in milliseconds for each individual trial, cognitive throughput on the STROOP incongruent trials and ADD were assessed by daily averages on the number of correct responses per minute, and inhibitory control on STROOP was calculated as the difference between incongruent reaction time and daily mean congruent reaction time. To eliminate any learning effect throughout the study, each metric was transformed to its z-score by subtracting the daily mean of all students and dividing it by the daily SD of all students. For both tests, an increase in reaction time z-score and a decrease in throughput z-score represent a deterioration in cognitive function. No other cognitive tests were administered to the students.

STROOP and ADD trials were identical and were presented in the same order to all students on a given study day, but they varied from day to day while keeping the proportion of congruency and mathematical operands constant (S1 Fig). The cognitive tests were developed as JavaScript testing engines for the Qualtrics online survey environment (Qualtrics, Provo, UT). Other researchers have demonstrated the usefulness of JavaScript-based online reaction time test engines [31]. The engines have chronometry and answer-grading capabilities embedded. To reduce the probability of timing errors due to varying communication speeds with the survey environment server, a fixed delay was introduced between consecutive trials. A neutral legend at the center of the screen was shown as a fixation point during the delay period.

The daily survey also included questions related to the past 24 hours on the following items: indoor environmental quality, sleep quality, intake of liquids and caffeinated beverages, and light exposure from electronic devices after 8 PM.

### Hydration and subjective sleepiness log

Daytime hydration was obtained via a text messaging server (Twilio, San Francisco, CA) asking for the liquid intake in number of glasses of water in the past 4 hours, and self-reported sleepiness was obtained using the Karolinska Sleepiness Scale (KSS) at 12 PM, 4 PM, and 8 PM each day. In an effort to standardize the hydration reporting units, students received a visual conversion scale to equate common glass and water bottles to number of glasses of approximately 8 fluid ounces (296 mL). A categorical variable based on the median daily water consumption (1 = less than or equal to 1 L/day; 0 = more than 1 L/day) was used as a surrogate for individual hydration. The KSS consists of a 9-point Likert scale used to subjectively assess the student’s sleepiness at the time of questioning [32]. The same questions for hydration and sleepiness were asked in the daily survey right after completion of the cognitive tests.

### Environmental and physiological measures

An indoor environmental quality monitor (Netatmo, Paris, France) was installed in each student’s bedroom. The monitor measured indoor dry-bulb temperature (°C), relative humidity (%), CO_2_ concentration (ppm), and noise (dBa). The monitors were installed by the study team in a place away from heat sources (e.g., computer screen, direct solar radiation, etc.) and sources of draft. Before deployment, CO_2_ was referenced to 400 ppm outdoor air to eliminate a drift error. CO_2_ drift and gain errors during deployment were estimated by collocating the IEQ monitors next to a recently calibrated instrument (Q-trak 7575; TSI Instruments, Shoreview, MN) inside a chamber, following 10 step-wise increments from 400 to 3,000 ppm. Values from the calibrated instrument were used as a reference to produce monitor-specific adjustment curves to match the experimentally derived values. There are many variables available to assess humidity [33], and as an attempt to utilize a mass-based representation of moisture in the air, indoor values of absolute humidity were derived from the measurements of relative humidity via the Ideal Gas Law. Hourly outdoor weather variables were obtained from the local airport weather station, located approximately 5 miles away from the study site.

Students wore an actigraphy-based sleep tracker (Basis Peak watch; Intel, Santa Clara, CA) on their nondominant wrist and were instructed to wear it at all times, especially during their sleep time. Total sleep time (TST) was estimated as the difference between sleep waking time and sleep onset time minus the interrupted sleep time quantified by the tracker. The tracker used photoplethysmography to measure heart rate (HR) in beats per minute (bpm) at a 1-minute resolution. The tracker has proven to be most accurate when collecting measurements at rest [34,35]. To avoid measurement noise introduced by student movement, we only considered HR measurements during night sleeping periods.

### Statistical analysis

To investigate the effect of having AC during the HW on cognitive function, we conducted a difference-in-differences (DiD) analysis, a method that emulates an experimental design by comparing the effect between exposure groups assuming that any difference between them would have remained constant had the intervention (AC) not taken place [36]. Considering having AC during a HW as a natural intervention, we used generalized linear mixed-effect models with each of the 5 cognitive performance metric z-scores as the outcome. First, we used an interaction term between heat exposure groups (exposed, non-AC = 1; unexposed, AC = 0) and an indicator variable signaling the start of the HW (baseline = 0; HW = 1) to estimate the cognitive effects among the non-AC group after the start of the HW. A variant of this model used an interaction for exposure group and the day difference to the beginning of the HW (July 14) to study the temporal trend in cognitive function changes. The coefficient of the interaction term is the resulting DiD estimate. We also accounted for 1-day lag maximum daily outdoor temperature. Student ID was specified as the random intercept accounting for the repeated measurements within subject.

Generalized additive mixed models were used to estimate the individual effects of indoor environmental parameters on cognitive function. Student ID, nested within building type, was treated as a random effect to account for differences between individuals. Environmental exposures to maximum indoor temperature, mean noise, mean absolute humidity, and mean CO_2_ concentrations were computed for the overnight period prior to each cognitive test and included into a single model for each cognitive function outcome of interest. In addition to indoor environmental exposures, we adjusted for hydration (glasses per day less than the median = 1; otherwise 0), caffeine intake (more than one caffeinated drink = 1; otherwise 0), and time from waking up to taking the test (time in hours). Nonlinear effects of continuous variables were evaluated by the use of penalized splines; only variables that exhibited a significant nonlinear effect were kept in the model as spline terms. In the case of finding a nonlinear relationship between cognitive function and indoor temperature, effect estimates were calculated by grouping the exposure variable (temperature) by quartiles. All model results show that residuals were normally distributed and homoscedastic. Because nighttime thermal exposure was the particular focus of the study, we wanted to understand the potential intermediate role of sleep in the causal pathway between temperature and cognitive function. We formulated a mediation model in which indoor temperature represented the exposure, TST represented the mediator, and the 5 cognitive metrics were the outcomes of interest. We used the “mediation” package from the R statistical software [37], which requires a 2-equation system of the outcome and mediator model as input. The results include the average causal mediator effect, the direct effect, and the total effect of the exposure. The same personal and behavioral covariates included in the environmental model described above were included in the outcome and mediator models.

All statistical analyses were completed in RStudio (version 1.1.414). Mann-Whitney-Wilcoxon tests were used to test building-level characteristics. We set a threshold for statistical significance at *p <* 0.05 for the main analyses (2-tailed tests). We present the results for all the outcomes and interpret the findings based on the consistency of the observed patterns, along with the magnitude and precision of effect estimates, rather than solely relying on statistical significance.

## Results

### Descriptive analyses

**Fig 1:**
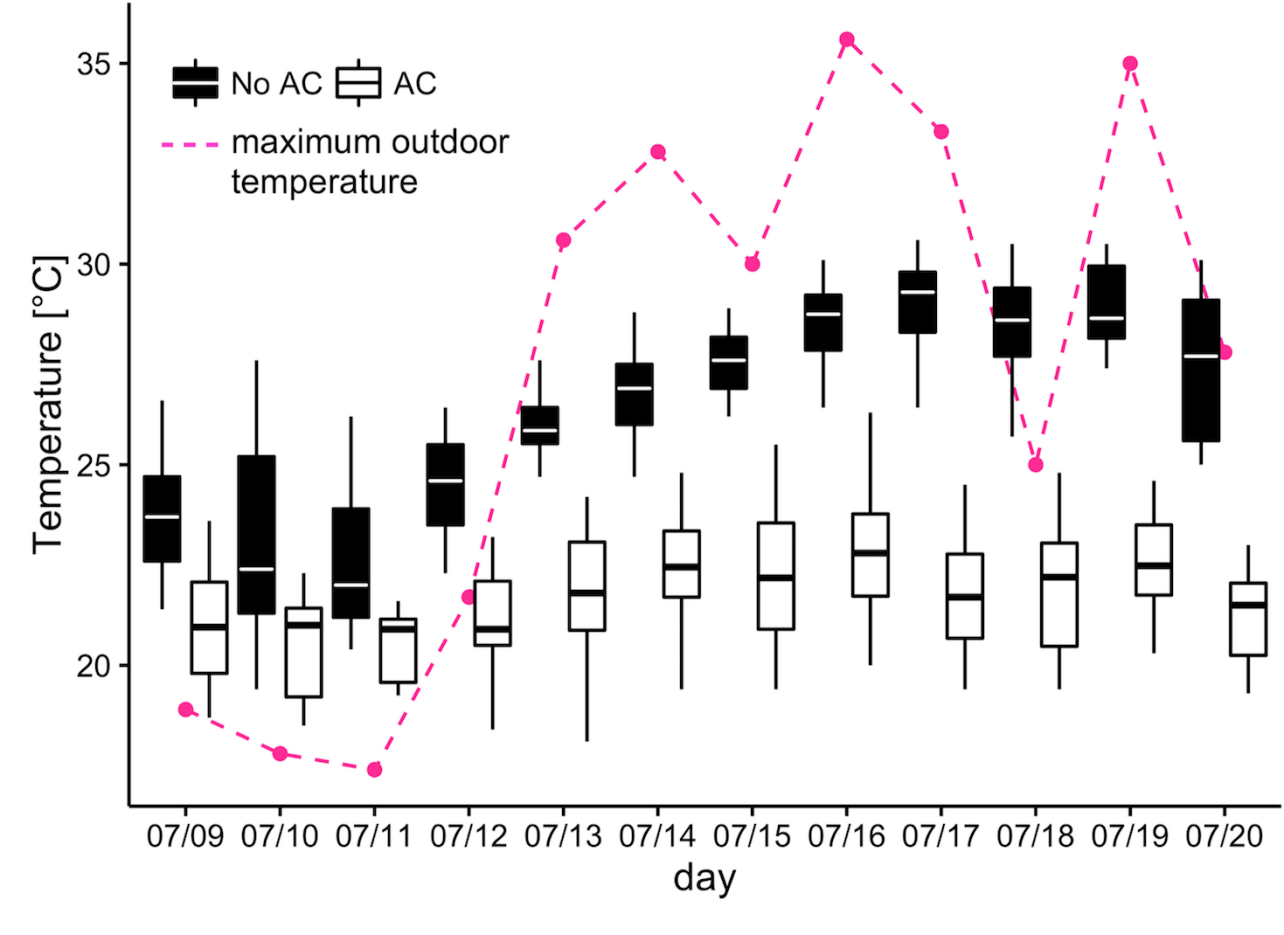
Indoor temperature distribution by exposure group (boxplots); 1-day lag maximum daily outdoor temperature (dotted line). AC, air conditioning.

Throughout the study, mean indoor temperatures in the non-AC group (mean = 26.3°C; SD = 2.5°C; range = 19.6–30.4°C) were significantly higher (*p <* 0.001) than in the AC group (mean = 21.4°C; SD = 1.9°C; range = 17.5–25.0°C) (Fig 1). The mean relative humidity in the non-AC group was significantly lower, at 61.4% (SD = 10.1%, range = 36%–88%), than in the AC group, for which it was 73.3% (SD = 7.4%; range = 48%–91%) (*p <* 0.001). Conversely, because air is dehumidified by the air-handling unit, absolute humidity was significantly lower (*p <* 0.001) in the AC group (14.1 g water/m^3^ air) than in the non-AC group (16.0 g water/m^3^ air). Mean CO_2_ levels were significantly lower in the non-AC group at 774.3 ppm (SD = 337.6 ppm; range = 366–1,688 ppm) compared to 1,667.5 ppm (SD = 783.6 ppm; range = 444–3,489 ppm) in the AC group (*p <* 0.001). Mean noise levels were significantly higher (*p <* 0.001) in the non-AC group, at 55.6 dBa (SD = 9.0 dBa; range = 38.0–76.9 dBa), compared to 46.2 dBa (SD = 7.1 dBa; range = 35.2–64.7 dBa) in the AC group. Indoor temperature exposure quartiles are 21.7°C, 23.5°C, and 26.9°C.

**Fig 2:**
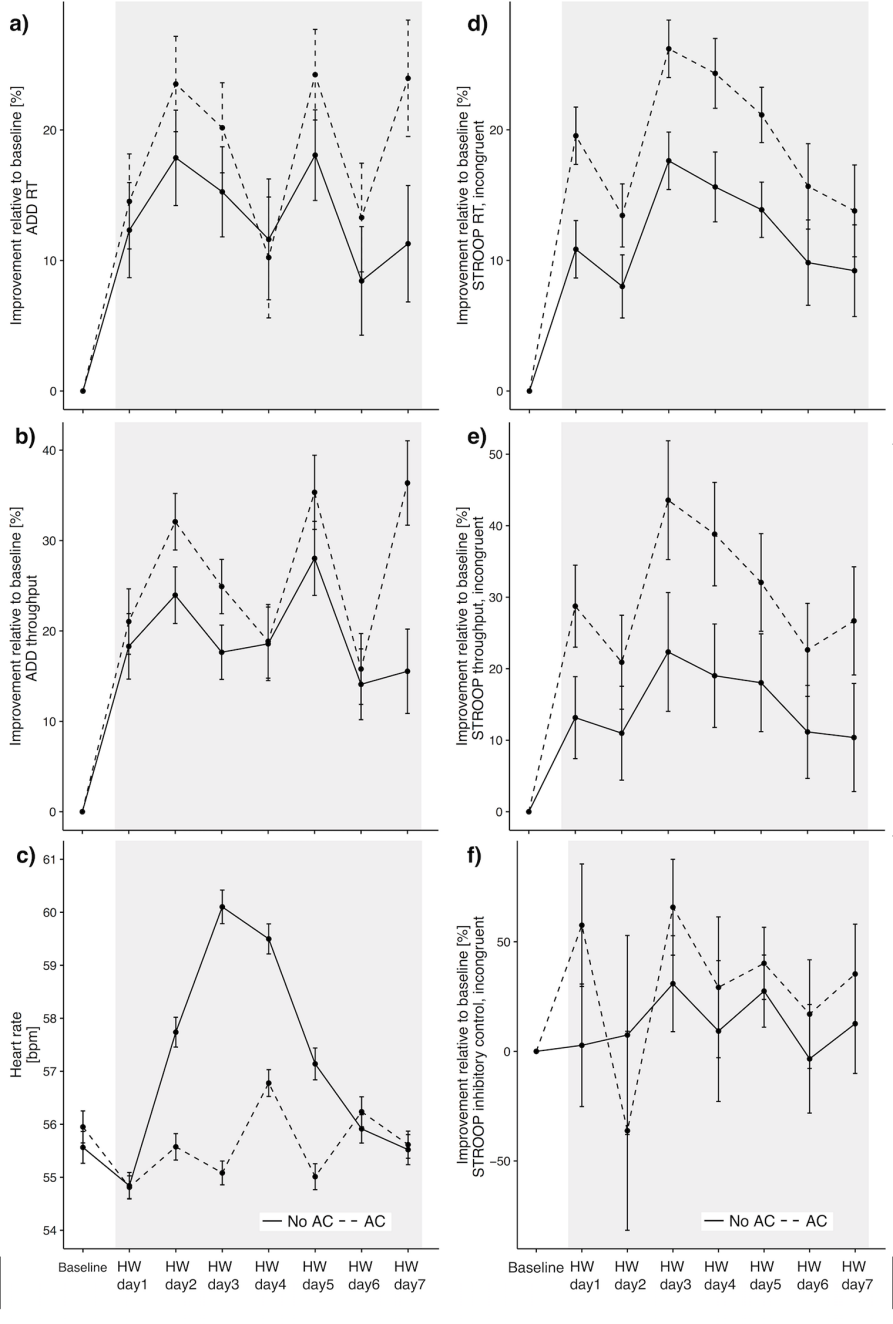
Individual improvement relative to baseline on STROOP and ADD performance (2a, 2b, and 2d–2f) and HR response (2c); error bars indicate 95% CI. AC, air conditioning; ADD, a 2-digit visual addition/subtraction test; bpm, beats per minute; HR, heart rate; HW, heat wave; RT, reaction time; STROOP, the Stroop color-word test.

Fig 2 shows the average individual improvement on STROOP and ADD performance relative to the 5-day baseline period. Due to the learning effect, both groups experienced an improvement during the course of the study. However, the AC group had significantly larger improvements in the 5 cognitive metrics. No significant difference at baseline was found between groups on ADD test metrics. Difference in STROOP inhibitory was also not significant. The AC group had significantly slower STROOP reaction time and lower STROOP throughput (S1 Table). Additionally, no significant difference in total number of daily steps was found between building types or within building type between baseline and HW. While absolute step count—as assessed by the physical activity tracker—was higher among non-AC students, this group registered a decrease of 467 steps per day during the HW versus a moderate increase (239 steps/day) registered by AC students (S2 Table). These estimations took into account the daily wear time of the device by each student, to avoid errors introduced by differential wear times (mean daily wear time: non-AC = 1,249 min/day; AC = 1,220 min/day).

### DiD results

To reduce the influence of a learning effect, cognitive test metrics were converted to z-scores to compare day-to-day cognitive test results in the DiD models. Results from the DiD pre- and post-HW models show significant deficits in both cognitive tests among the non-AC group after the onset of the HW, relative to the AC group (Table 2). The largest effects were observed in STROOP. STROOP throughput in the non-AC group had a mean difference in z-score from baseline of −0.53 (*p =* 0.0001), equivalent to a reduction in 4.8 rpm or a 9.91% performance decrement with respect to the AC group. In the non-AC group, STROOP reaction time and STROOP inhibitory control increased by 155 ms (13.4%) and 22 ms (13.3%), respectively, versus the AC group. ADD reaction time in the non-AC group had a mean z-score increase of 0.12 (*p =* 0.0001) with respect to the AC group, equivalent to 288 ms (11.4%). ADD throughput also exhibited the same trend, though not significantly between the exposure groups during the HW (mean difference in z-score = −0.19; *p =* 0.08), equivalent to 0.97 rpm—or a 6.3% performance decrement less with respect to the AC group.

**Table 2:** Effects of HWs on cognitive performance in STROOP and ADD. DiD estimates of cognitive tests in non-AC group compared to AC group; mean difference in z-score from baseline (95% CI).

	ADD		STROOP			
	Reaction time (*n =* 44; obs = 3,766)	Throughput (*n =* 44; obs = 404)	Reaction time (*n =* 44; obs = 4,225)	Throughput (*n =* 44; obs = 418)	Inhibitory control (*n =* 44; obs = 4,225)	T_out,max_ (°C) 1-day lag
**Pre–post HW model**						
Intervention	0.12*** (0.05–0.18)	−0.19 (−0.44 to 0.05)	0.35*** (0.23–0.46)	−0.53*** (−0.78 to −0.27)	0.16* (0.02–0.29)	
**Day from start of HW model**						
HW day 1	0.07 (−0.04 to 0.18)	−0.03 (−0.45 to 0.40)	0.43*** (0.62–0.25)	−0.56* (−1.02 to −0.10)	0.30** (0.09–0.51)	32.8
HW day 2	0.11 (−0.01 to 0.21)	−0.27 (−0.68 to 0.15)	0.27** (0.09–0.45)	−0.39 (−0.85 to 0.04)	−0.03 (−0.17 to 0.23)	30
HW day 3	0.09 (−0.02 to 0.20)	−0.2 (−0.62 to 0.23)	0.46*** (0.28–0.64)	−0.69** (−1.15 to −0.23)	0.35** (0.07–0.47)	35.6
HW day 4	0.06 (−0.05 to 0.17)	−0.12 (−0.54 to 0.29)	0.49*** (0.25–0.72)	−0.69** (−1.14 to −0.23)	0.27** (0.10–0.61)	33.3
HW day 5	0.16** (0.06,0.27)	−0.25 (−0.66 to 0.16)	0.40*** (0.22–0.58)	−0.45* (−0.99 to −0.01)	0.04 (−0.16 to 0.24)	25
HW day 6	0.13* (0.02–0.24)	−0.16 (−0.25 to 0.58)	0.37** (0.13–0.60)	−0.46* (−1.01 to −0.01)	0.06 (−0.20 to 0.32)	35
HW day 7	0.18** (0.06–0.29)	−0.47* (−0.92 to −0.01)	0.22 (−0.04 to 0.46)	−0.43 (−0.98 to 0.00)	0.23 (−0.04 to 0.49)	27.8

Significance levels at

*p < 0.05

**p < 0.01

***p < 0.001.

Abbreviations: AC, air conditioning; ADD, a 2-digit visual addition/subtraction test; DiD, difference-in-differences; HW, heat wave; obs, number of observations; STROOP, the Stroop color-word test; T_out,max_, daily maximum outdoor temperature.

Changes in the magnitude and significance of DiD estimates in the day from start of the HW model show different temporal trends by cognitive domain (Table 2). On one hand, the deficits in the non-AC group in ADD reaction time and throughput increased progressively along the HW period. The effects became significant towards the end of the HW, despite the outdoor temperature reductions on days 5 and 7 of the HW period (Table 2). These effects were likely driven by indoor temperature levels that prolonged the heat exposure beyond the official HW period according to its definition based on outdoor weather parameters. On the other hand, the largest and most significant effects in the STROOP reaction time, throughput, and inhibitory control time were observed during the days following the highest daily T_out,max_ (i.e., HW days 1, 3, 4, and 6). In contrast to ADD performance, the effect in STROOP was reduced in magnitude and insignificant for reaction time (mean difference in z-score = 0.22; *p =* 0.08) and throughput (mean difference in z-score = −0.43; *p =* 0.08) at the end of the HW.

A similar trend was observed in measured HR during sleep periods, as categorized by the sleep tracker. Fig 2C shows a significant increase in HR among the non-AC group during the hottest days of the HW (mean difference: 2.39 bpm; 95% CI 2.28–2.56 bpm), but it receded in the last 2 days of the study.

### Effects of indoor environmental factors

**Fig 3:**
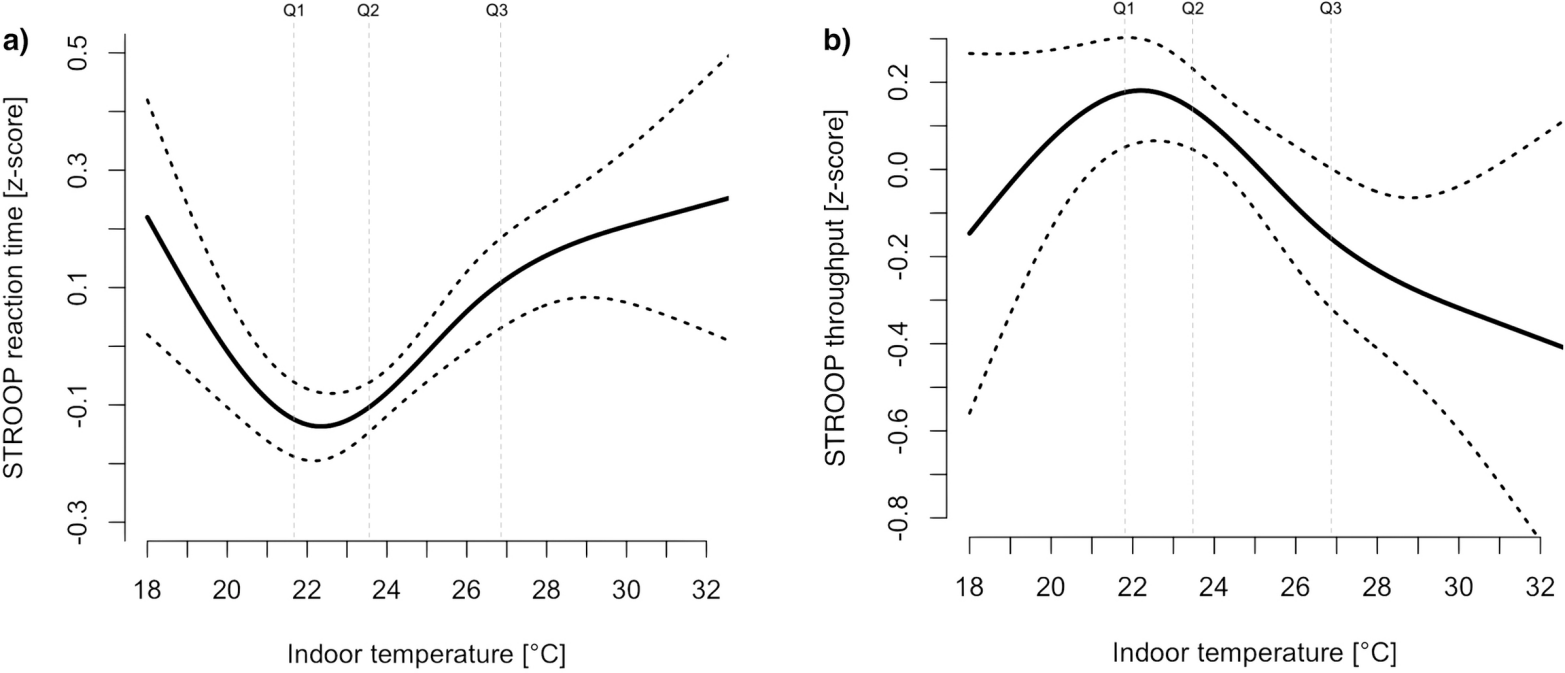
CS relationships between maximum indoor temperature and (a) STROOP reaction time and (b) STROOP throughput predicted from the fitted environmental exposure models in Table 3. CS, cubic spline; STROOP, the Stroop color-word test.

Maximum indoor temperature during the sleep period prior to the daily cognitive testing was significantly associated with the performance on the 5 cognitive function metrics studied here (Table 3). For ADD reaction time, ADD throughput, and STROOP inhibitory control time, performance decreased linearly with an increase in indoor temperature exposure. For the mean difference in indoor temperatures between exposure groups during the HW (6.51°C; 95% CI 6.48–6.53), the estimated mean difference in z-score is 0.06 (95% CI 0.02–0.12) for ADD reaction time, −0.24 (95% CI −0.52 to −0.01) for ADD throughput, and 0.18 (95% CI 0.03–0.26) for STROOP inhibitory control time. The association between indoor temperature exposure and STROOP reaction time and STROOP throughput was described by a U-shaped (Fig 3A) and an inverse U-shaped relationship (Fig 3B), respectively. An optimum in STROOP reaction time and STROOP throughput was found at approximately 22°C; deviations from this value in either direction resulted in a performance decline. In the range between 22°C and 28°C, the approximate linear effects of temperature on z-score are 0.04 per°C and 0.05 per°C in STROOP reaction time and STROOP throughput, respectively. Additionally, Table 3 shows the estimated effects of indoor temperature grouped by exposure quartiles.

**Table 3:** Summary of fixed-effect estimates from the environmental exposure models, adjusting for personal characteristics, sleep, and behavioral factors. Cognitive tasks fixed-effect estimates, mean difference in z-score (95% CI).

	ADD		STROOP		
	Reaction time	Throughput	Reaction time	Throughput	Inhibitory control time
**Environmental factors**					
Indoor temperature [°C]	0.01* (0.003–0.02)	−0.04* (−0.08 to −0.002)	Q2 = −0.09 (−0.19 to 0.01)	Q2 = 0.17 (−0.05 to 0.39)	0.03* (0.005–0.05)
			Q3 = 0.02 (−0.15 to 0.10)	Q3 = −0.02 (−0.28 to 0.24)	
			Q4= 0.23** (0.06–0.38)	Q4= −0.32* (−0.64 to −0.01)	
CO_2_ [ppb]	−0.006 (−0.04 to 0.02)	−0.06 (−0.17 to 0.06)	0.01 (−0.05 to 0.07)	−0.001 (−0.13 to 0.13)	CS** (*p =* 0.01)
Noise [dBa]	−0.002 (−0.001 to 0.006)	−0.01 (−0.02 to 0.003)	−0.01*** (−0.02 to −0.005)	0.01* (0.001–0.03)	−0.006* (−0.01 to −0.0001)
Absolute humidity [g water/m^3^ air]	0.003 (−0.01 to 0.01)	0.001 (−0.04 to 0.03)	−0.0003 (−0.02 to 0.02)	−0.001 (−0.04 to 0.04)	−0.02 (−0.04 to 0.01)
**Personal characteristics**					
Gender [Women]	0.08 (−0.05 to 0.22)	−0.36 (−0.83 to 0.10)	−0.02 (−0.30 to 0.25)	0.02 (−0.47 to 0.51)	−0.05 (−0.21 to 0.10)
Age [Years]	0.04* (0.01 to 0.08)	−0.11 (−0.24 to 0.01)	0.13*** (0.05–0.20)	−0.17* (−0.30 to −0.04)	0.01 (−0.04 to 0.05)
Born in US	−0.02 (−0.17 to 0.13)	0.15 (−0.36 to 0.66)	0.02 (−0.28 to 0.32)	−0.12 (−0.65 to 0.41)	0.05 (−0.12 to 0.22)
**Behavioral factors**					
Caffeine intake [>1 drink]	−0.04* (−0.08 to −0.001)	0.13 (−0.001 to 0.30)	−0.07 (−0.14 to 0.01)	0.13 (−0.04 to 0.30)	−0.11** (−0.19 to −0.03)
Liquid intake [<6 glasses per day]	0.14* (0.01–0.27)	−0.46* (−0.91 to −0.01)	0.12 (−0.14 to 0.38)	0.23 (−0.23 to 0.70)	0.08 (−0.07 to 0.23)
Time to test after awake [Hours]	−0.004 (−0.01 to 0.004)	0.02 (−0.01 to 0.05)	−0.002 (−0.02 to 0.01)	−0.004 (−0.03 to 0.04)	0.05* (0.003–0.10)

Significance levels at

*p < 0.05

**p < 0.01

***p < 0.001.

Abbreviations: ADD, a 2-digit visual addition/subtraction test; CS, cubic spline; ppb, parts per billion; STROOP, the Stroop color-word test.

In addition to temperature, noise had an arousal effect on STROOP reaction time and STROOP inhibitory control. A nonlinear association was found between CO_2_ levels and inhibitory control time. When dichotomizing the CO_2_ concentrations according to the median value of the study (median CO_2_ = 1,250 ppm), a CO_2_ concentration above the median was associated with slower STROOP inhibitory control time (mean difference in z-score = 0.13; 95% CI 0.01–0.25; *p =* 0.02) (Table 3).

Personal characteristics and behavioral factors showed significant effects on cognitive function (Table 3). Individuals with a liquid intake below the mean (<6 glasses per day) had significant deficits in ADD. Conversely, drinking more than one caffeinated beverage per day had an arousal effect on both ADD metrics and STROOP inhibitory control time. A significant effect of the lapse duration between wake-up time to taking the test was found for only STROOP inhibitory control time.

### Mediation analysis

An increase in 1°C in overnight indoor temperature exposure resulted in a 2.74-minute decrease in TST (95% CI −2.77 to −2.71; *p <* 0.001). The mediation analysis revealed a significant mediation effect of TST for ADD reaction time, representing 8.4% of the total effect (S3 Table). The direct effect of temperature was predominant on the 5 cognitive metrics. A nonsignificant mediation effect of TST on STROOP shows that longer sleep times are associated with shorter reaction time and increased throughput.

## Discussion

We found that individuals in non-AC buildings experienced reductions in cognitive function, as assessed by working memory and selective attention/processing speed, ranging from 4.1% to 13.4% relative to baseline and with respect to the AC group. The analysis suggests that these reductions might be attributable to an increase in thermal load and the combined influence of other environmental (e.g., ventilation, acoustics) and behavioral (e.g., hydration, sleep) factors that compound the effects of heat exposure in real-life settings. Increasing evidence from experimental, epidemiological, and econometric studies has demonstrated the effects of increased heat exposures on productivity [38], learning ability [39], and morbidity and mortality in humans [17,18,40]. This research builds upon this body of evidence because it is the first field study demonstrating the detrimental cognitive effects of a HW in a group of young, healthy individuals. Research focusing on the mechanistic pathways in which body temperature modulates neurobehavioral function in humans often relies on the experimental inducement of passive hyperthermia via controlled environmental exposure to heat. These efforts, however, fail at recreating the complex environmental and behavioral factors influencing cognitive function found in real settings. In contrast, we were able to comprehensively characterize the environmental exposures and physiological reactions as well as behaviors of 2 groups drawn from the same population but with differential heat exposure during a naturally occurring HW. Therefore, the environmental exposures are of unequivocal validity in terms of magnitude, duration, and complexity.

### Relationship between indoor temperature and cognitive function

Analysis of the individual effects of indoor environmental parameters indicate that higher indoor temperatures during the sleep period resulted in significant cognitive function deficits across the 5 cognitive metrics considered in this study. Our findings support previous reports of relative throughput reductions in ADD (−11.7%; *p =* 0.01) and STROOP (−9.5%; *p =* 0.09) at 30°C compared to 22°C [27]. Similarly, we found a 9.51% relative reduction in ADD throughput and 7.48% reduction in STROOP throughput for an equivalent temperature exposure (Δ8°C). We found a U-shaped and inverted U-shaped relationship between indoor maximum temperature overnight and STROOP reaction time and throughput, respectively. Similar nonlinear relationships between indoor temperature and cognitive test performance show an optimum range for cognitive tests and office work productivity centered around 22°C [27,41]. This temperature value is lower than the neutral thermal point (26 ± 0.5°C) predicted by the physiology-based heat exchange model proposed by Gagge and Nishi (2011) for a young population in a light summer clothing envelope, as well as the adaptive thermal comfort model [42].

By comparison, STROOP inhibitory control reaction time, as well as ADD reaction time and throughput, exhibited a linear relationship with indoor temperature exposures, suggesting that arousal temperature for inhibitory control and working memory might have some lower optima than working speed in STROOP. It has been previously proposed that differential effects to the various cognitive domains might result from zone-specific thermal sensitivities in the brain [43]. Sun and colleagues (2013), for example, found an increase in inhibitory control response time among subjects passively induced to hyperthermia; they attributed this effect to significant changes in the functional connectivity of brain areas responsible for high-order executive function and somatosensory signal transmission of skin temperature stimuli (decreased connectivity of the medial orbitofrontal cortex and temporal and parietal lobes) and thermoregulation (increased connectivity of hypothalamic areas) [26]. Others have found a performance impairment in ADD at different increments of core temperature induced by controlled heat exposure [44]. Analog to their findings, we also observed a progressive performance deficit associated with heat exposure, despite the suggestive evidence that subjects had reached some level of physiological adaptation by the end of the study. In our case, the non-AC group showed a significant increase in HR during sleep for the first days of the HW, followed by a reduction to pre-intervention HR levels during the last 2 days of the study, although indoor temperatures during those days remained virtually unchanged (Fig 2C). In ADD, we found a linear relationship between indoor temperature and cognitive function deficits. Cognitive deficits due to cold have been found at lower temperatures than those we observed [20,44], which might explain why our findings did not show an inflection point in ADD performance.

### Relationship between other environmental exposures and cognitive function

Differences in environmental exposures among the AC and non-AC groups extended beyond temperature. Low ventilation rates in the AC group led to significantly higher CO_2_ concentrations during sleep periods (*p <* 0.001). Low ventilation rates and CO_2_ concentrations normally found in office buildings (approximately 1,000 ppm) have been recently associated with impairment in multiple domains of high-order cognitive function [45,46]. We found that CO_2_ concentrations beyond the median (1,250 ppm) were associated with longer inhibitory reaction times (z-score = 0.13 ± 0.06; *p =* 0.02). The predominant role of the temperature exposure might explain why no other cognitive test metrics were significantly associated with CO_2_ concentrations [27], as found by Lan and colleagues (2017). Significantly higher noise levels in the exposed group were likely due to the continuous use of fans and open windows as a heat-mitigation strategy during the HW. A significant improvement in reaction time and throughput in STROOP was associated with higher decibel levels, which is consistent with previous findings of antagonistic effects of noise and temperature exposures [47,48]. An early explanation of this phenomenon was proposed by Houston (1969) [49] based on the effect of noise inhibiting the interference of other types of stimuli (e.g., temperature, incongruent color-word), thus facilitating the performance of the evaluated task.

### Relationship between behavioral factors and cognitive function

In the current study, we used self-reported liquid intake as an indirect measure of hydration level. Despite the limitations of this indicator, we found that an intake below the median in the study (i.e., 6 glasses of liquid per day) was associated with cognitive function deficits in all evaluated metrics. Using more precise markers of dehydration, others have found that cognitive function impairment is associated with hypovolemia, further compounded by heat stress and physical activity [23,50]. STROOP inhibitory control was sensitive to the lapse between wake-up and test time, with poorer performance associated with longer lapse times. In 2015, Burke and colleagues [51] reported impaired performance right after wake-up, gradually improving in the following 2- to 4-hour period as sleep inertia dissipates. Because the mean lapse time in our study was 0.9 hours (SD = 0.81), the effects of sleep inertia might have still impacted this cognitive outcome.

Results from the mediation analysis suggest that sleep might be an intermediate variable in the causal mechanism between indoor temperature exposures and cognitive function, which were significant for ADD throughput. Our small sample size and limited reliability of the actigraphy-based TST values preclude this analysis from yielding conclusive results about the mediating role of sleep in cognitive effects.

### Implications for existing adaptation strategies to extreme heat in buildings

Our findings have relevant implications for building design, redesign, and adaptation strategies to a changing climate. The results from the DiD models suggest that the HW had a causal detrimental effect on 5 cognitive function measures in the exposed group (non-AC) with respect to the control group (AC) during the baseline period. The relative magnitude of these effects is substantial as well. The relative reduction of 13.9% (z-score = −0.47) in ADD throughput on the last day of the HW is comparable to the 15.8% relative reduction in the throughput on a serial ADD associated with 24 hours of sleep deprivation [52]. A closer look at the temporal trend of the effects, particularly in ADD, shows a progressively increasing difference in cognitive function between the 2 groups; in fact, the effect estimates are largest after outdoor temperature values have subsided. However, the large thermal inertia characteristic of buildings in heating-dominated climates prolonged high indoor heat exposures beyond the official duration of the HW (Fig 1). Because AC systems are often an addendum to existing construction, the building structural properties are similar to their non-AC counterparts, and therefore their protective effect is contingent upon electricity availability. The implications associated with the limited passive habitability of the current built environment have been reported before [53,54]. Our results stress the need to account for the building thermal properties that modify indoor heat exposure. Current building simulation tools might be used to model the thermal retention potential of building structures to compute a high–spatial-resolution indoor-heat index prediction. Such tools could be implemented at the city level as part of extreme heat adaptation plans.

These results provide evidence to mitigate heat exposures among populations normally considered resilient to them, especially in settings in which cognitive processes are critical to ensure learning, safety, or productivity. The increased adoption of current mechanical cooling systems, however, entails several unresolved challenges. First, their use represents a positive feedback to climate change due to the increased greenhouse gas emissions associated with higher energy demand and unintended refrigerant leaks, even in locations where renewables constitute a larger portion of the electricity generation mix [55–57]. Second, their deficient design and operation, often leading to overcooled and underventilated spaces, have detrimental effects on cognitive function and increase the exposure risk to indoor pollutant sources [58]. Finally, long-term exposures to thermally controlled environments might have a maladaptive effect on physiological acclimatization, potentially increasing the biological susceptibility to heat stress [59]. Novel building materials [60] might represent alternative solutions for the uncompromised management of more demanding extreme thermal regimes expected in the future.

The students’ limited age range represents a limitation in our study. Nevertheless, the consistent findings in this young, healthy population might indicate that greater portions of the population are equally or more susceptible to these effects. Another limitation is that the study was performed in a heating-dominated climate, which could compromise the generalizability of the results. Further research in other latitudes and settings should address the question of susceptibility and acclimatization to heat stress. Because the daily cognitive assessments took place right after waking, our study examines acute affects that may be transitory in this or other populations. Lacking information on location and concurrent environmental exposures during the day, we could not determine whether the observed effects extend during the rest of the day. However, we consider that cognitive function demands during the earliest time window involve critical tasks, such as commuting, that have been found to be impacted by HWs [61]. Further research could examine the prolonged effects of this association during the day. Air pollution may increase during HWs, resulting in different indoor concentrations if AC usage or window use patterns differ between buildings. While negative effects of air pollution on cognitive function are mostly attributed to neurodevelopmental changes [62], recent evidence also suggests an acute effect of criteria pollutants such as nitrogen dioxide and elemental carbon on attention processes [63].

Health effects of heat stress due to climate change, manifested as cognitive function deficits, extend to larger sectors of the population and can have significant implications on educational attainment, economic productivity, and workplace safety. Indoor temperature exposures in the non-AC buildings prolonged the official HW period. Given the importance of indoor heat exposures, we deem it necessary to consider building thermal properties in indoor heat indices when forecasting HWs for the proper assessment of heat exposure risks. Moreover, existing methods to mitigate indoor heat exposures only provide a short-term solution by means of localized thermal comfort at the expense of a potential increase in greenhouse gas emissions and higher exposures to indoor contaminants. A sustainable management of indoor heat loads will require the use of novel building design and materials, as well as scalable technological advancements in building ventilation and cooling systems.

## Supporting information

S1 STROBE Checklist (DOCX)

S1 Table Difference in test performance at baseline, z-score. (DOCX)

S2 Table Total number of steps measured by physical activity tracker. (DOCX)

S3 Table Effect estimates from mediation model of TST (mediator) and indoor temperature (exposure) on cognitive tests, mean difference in z-score (95% CI). TST, total sleep time.
(DOCX)

S1 Fig Examples of daily STROOP and ADD electronic tests shown in smartphone display. ADD, a 2-digit visual addition/subtraction test; STROOP, the Stroop color-word test.
(DOCX)

## Abbreviations

AC: air conditioning
ADD: a 2-digit visual addition/subtraction test
bpm: beats per minute
CS: cubic spline
DiD: difference-in-differences
HR: heart rate
HW: heat wave
KSS: Karolinska Sleepiness Scale
NOAA: National Oceanic and Atmospheric Administration
ppb: parts per billion
STROOP: the Stroop color-word test
T_out,max_: daily maximum outdoor temperature
TST: total sleep time
