# Supplementary material for: Reduced cognitive function during a heat wave among residents of non-air-conditioned buildings: An observational study of young adults in the summer of 2016
Source: PLoS Med. 2018 Jul 10;15(7):e1002605. doi: 10.1371/journal.pmed.1002605 (PMC6039003; doi:10.1371/journal.pmed.1002605)
Supplement: S1 STROBE Checklist — (DOCX) [file pmed.1002605.s001.docx]

STROBE Statement—checklist of items that should be included in reports of observational studies

|  | Item No | Recommendation | Addressed in Manuscript |
| --- | --- | --- | --- |
| **Title and abstract** | 1 | (*a*) Indicate the study’s design with a commonly used term in the title or the abstract | Included in the abstract, second paragraph |
|  |  | (*b*) Provide in the abstract an informative and balanced summary of what was done and what was found | Included throughout abstract, second and third paragraph |
| Introduction | | |  |
| Background/rationale | 2 | Explain the scientific background and rationale for the investigation being reported | Included throughout introduction, first paragraph |
| Objectives | 3 | State specific objectives, including any pre-specified hypotheses | Introduction, last paragraph |
| Methods | | |  |
| Study design | 4 | Present key elements of study design early in the paper | Methods, Study design section, |
| Setting | 5 | Describe the setting, locations, and relevant dates, including periods of recruitment, exposure, follow-up, and data collection | Highlighted in methods sub-section ‘Study Design’, first and second paragraph |
| Participants | 6 | (*a*) *Cohort study*—Give the eligibility criteria, and the sources and methods of selection of participants. Describe methods of follow-up | Included in methods subsection ‘Study Participants’ paragraph two and Table 1 |
|  |  | (*b*) *Cohort study*—For matched studies, give matching criteria and number of exposed and unexposed | Not applicable, since this was not a “matched” study. However, we recruited balanced numbers of those living with and without AC Methods, second paragraph |
| Variables | 7 | Clearly define all outcomes, exposures, predictors, potential confounders, and effect modifiers. Give diagnostic criteria, if applicable | Outcomes: STROOP RT, STROOP throughput, STROOP inhibitory control, ADD RT, ADD throughput  Exposures: temperature, humidity, CO2, noise  Predictors: temperature and AC  Potential confounders: age, gender, caffeine intake, hydration, time from waking to test  Effect Modifiers: NA  Mediator: total sleep time (TST)  Found in Methods section, paragraphs 3-6 |
| Data sources/ measurement | 8* | For each variable of interest, give sources of data and details of methods of assessment (measurement). Describe comparability of assessment methods if there is more than one group | Each variable of interest is highlighted within a respective Methods subsection, including surveys, hydration and sleepiness logs, environmental measures, physiological measures, and the cognitive tests  Found in Methods section, paragraphs 3-6 |
| Bias | 9 | Describe any efforts to address potential sources of bias | The differences in differences approach was adopted to minimize potential confounding between treatment and control groups Statistical models included confounding variables known to this topic [Methods section, paragraph 7, Statistical analysis subsection]. |
| Study size | 10 | Explain how the study size was arrived at | Study size was limited by available study equipment and funding. Samples between building types were balanced during recruitment [Methods paragraph 1] |
| Quantitative variables | 11 | Explain how quantitative variables were handled in the analyses. If applicable, describe which groupings were chosen and why | Outcomes were normalized and z-scores were used in all statistical models. All environmental variables were used as continuous variables with no transformation. [Methods section, paragraph 7, Statistical analysis subsection] |
| Statistical methods | 12 | (*a*) Describe all statistical methods, including those used to control for confounding | Included throughout methods section [Methods section, paragraph 7, Statistical analysis subsection] |
|  |  | (*b*) Describe any methods used to examine subgroups and interactions | We controlled for gender and age. |
|  |  | (*c*) Explain how missing data were addressed | Missing sleep data from physical activity monitor was imputed from survey data. |
|  |  | (*d*) *Cohort study*—If applicable, explain how loss to follow-up was addressed | No loss to follow-up was experienced. |
|  |  | (*e*) Describe any sensitivity analyses | NA |

Continued on next page

| Results | | |  |
| --- | --- | --- | --- |
| Participants | 13* | (a) Report numbers of individuals at each stage of study—eg numbers potentially eligible, examined for eligibility, confirmed eligible, included in the study, completing follow-up, and analysed | Samples reported [lines 109-110] were consistent throughout each stage of study, as no participants who expressed interest were illegible and no loss to follow-up occurred [Results paragraph 1] |
|  |  | (b) Give reasons for non-participation at each stage | No participants were lost to follow-up. |
|  |  | (c) Consider use of a flow diagram | Since sample sizes were consistent from recruitment through the duration of the study, a flow diagram is not included. |
| Descriptive data | 14* | (a) Give characteristics of study participants (eg demographic, clinical, social) and information on exposures and potential confounders | Highlighted in Table 1 [Methods] and Figure 1 [Results, after paragraph 1] |
|  |  | (b) Indicate number of participants with missing data for each variable of interest | Temperature data was complete. |
|  |  | (c) *Cohort study*—Summarise follow-up time (eg, average and total amount) | Included in Methods subsection ‘Study Design’ [paragraph 1] |
| Outcome data | 15* | *Cohort study*—Report numbers of outcome events or summary measures over time | 5 outcomes of interest were recorded: STROOP RT, STROOP throughput, STROOP inhibitory control, ADD RT, ADD throughput [Methods, survey instruments, daily survey and cognitive test] |
| Main results | 16 | (*a*) Give unadjusted estimates and, if applicable, confounder-adjusted estimates and their precision (eg, 95% confidence interval). Make clear which confounders were adjusted for and why they were included | See Results, Table 3  Confounders were chosen based on plausible relation to the outcome from previous research. |
|  |  | (*b*) Report category boundaries when continuous variables were categorized | NA |
|  |  | (*c*) If relevant, consider translating estimates of relative risk into absolute risk for a meaningful time period | NA |
| Other analyses | 17 | Report other analyses done—eg analyses of subgroups and interactions, and sensitivity analyses | Mediation analysis was completed to assess the impact of total sleep time as a mediator between temperature exposure and cognitive function. [Results paragraph 9] |
| Discussion | | |  |
| Key results | 18 | Summarise key results with reference to study objectives | Main results included in Discussion [paragraph 1] |
| Limitations | 19 | Discuss limitations of the study, taking into account sources of potential bias or imprecision. Discuss both direction and magnitude of any potential bias | Included in Discussion [ paragraph 8] |
| Interpretation | 20 | Give a cautious overall interpretation of results considering objectives, limitations, multiplicity of analyses, results from similar studies, and other relevant evidence | Main results included in Discussion [paragraphs 1-4] as well as relevant evidence from similar studies throughout Discussion (paragraphs 1-6] |
| Generalisability | 21 | Discuss the generalisability (external validity) of the study results | Included in Discussion [paragraph 8] |
| Other information | | |  |
| Funding | 22 | Give the source of funding and the role of the funders for the present study and, if applicable, for the original study on which the present article is based | Harvard University Climate Change Solutions Fund 2015. Despite providing funding for this study, the university did not play a role in determining the study design, outcomes, or analysis. |

*Give information separately for cases and controls in case-control studies and, if applicable, for exposed and unexposed groups in cohort and cross-sectional studies.

**Note:** An Explanation and Elaboration article discusses each checklist item and gives methodological background and published examples of transparent reporting. The STROBE checklist is best used in conjunction with this article (freely available on the Web sites of PLoS Medicine at http://www.plosmedicine.org/, Annals of Internal Medicine at http://www.annals.org/, and Epidemiology at http://www.epidem.com/). Information on the STROBE Initiative is available at www.strobe-statement.org.
