# Supplementary material for: Reduced cognitive function during a heat wave among residents of non-air-conditioned buildings: An observational study of young adults in the summer of 2016
Source: PLoS Med. 2018 Jul 10;15(7):e1002605. doi: 10.1371/journal.pmed.1002605 (PMC6039003; doi:10.1371/journal.pmed.1002605)
Supplement: S1 Table — (DOCX) [file pmed.1002605.s002.docx]

Table S1 Difference in test performance at baseline, z-score

|  | | | |
| --- | --- | --- | --- |
| **ADD** | **non-AC** | **AC** | **p-value** |
| Reaction time | 0.02 | -0.01 | 0.18 |
| Throughput | -0.08 | 0.11 | 0.26 |
| **STROOP** |  |  |  |
| Reaction time | -0.08 | 0.1 | 0.001*** |
| Throughput | 0.17 | -0.17 | 0.03* |
| Inhibitory control | -0.05 | 0.05 | 0.07 |

*Significance level at * p< 0.05 ; ** p<0.01, *** p<0.001*
