# Supplementary material for: Reduced cognitive function during a heat wave among residents of non-air-conditioned buildings: An observational study of young adults in the summer of 2016
Source: PLoS Med. 2018 Jul 10;15(7):e1002605. doi: 10.1371/journal.pmed.1002605 (PMC6039003; doi:10.1371/journal.pmed.1002605)
Supplement: S2 Table — (DOCX) [file pmed.1002605.s003.docx]

Table S2. Total number of steps measured by physical activity tracker

|  | **Building type** | |  |
| --- | --- | --- | --- |
| **Average total daily steps (SD)** | Non-AC | AC | **Δ by building type  (p-value)** |
| Baseline | 10902 (5715) | 9384 (4987) | 1518 (0.06) |
| Heatwave | 10435 (5747) | 9623 (5152) | 812 (0.19) |
|  |  |  |  |
| **Δ by study phase (p-value)** | 467 (0.51) | -239 (0.77) |  |

*Significance level at * p< 0.05 ; ** p<0.01, *** p<0.001*
