# Supplementary material for: Reduced cognitive function during a heat wave among residents of non-air-conditioned buildings: An observational study of young adults in the summer of 2016
Source: PLoS Med. 2018 Jul 10;15(7):e1002605. doi: 10.1371/journal.pmed.1002605 (PMC6039003; doi:10.1371/journal.pmed.1002605)
Supplement: S3 Table — TST, total sleep time. (DOCX) [file pmed.1002605.s004.docx]

Table S3. Effect estimates from mediation model of TST (mediator) and indoor temperature (exposure) on cognitive tests, mean Difference in z-Score (95% CI)

|  |  | | | | | |  |
| --- | --- | --- | --- | --- | --- | --- | --- |
|  | **ADD** | | | **STROOP** | | |  |
|  | Reaction time | Throughput | Reaction time | | Throughput | Inhibitory control | |
| **Mediation analysis effects** |  |  |  | |  |  | |
| Total sleep time,  average causal mediation effect | **-0.001 (-0.002, -0.0004)***** | 0.003 (-0.002, 0.009) | -3.96e-6 (-0.001, 0.001) | | 0.0005 (-0.003, 0.004) | 0.0003 (-0.001, 0.001) | |
| Indoor temperature,  average direct effect per 1°C | **0.013 (0.001, 0.023)*** | **-0.04 (-0.081, -0.002)*** | **0.032 (0.011, 0.054)***** | | **-0.051 (-0.092, -0.007)*** | **0.021 (0.003, 0.041)*** | |
| Total Effect | **0.012 (0.003, 0.022)*** | -0.037 (-0.077, 0.002) | **0.032 (0.011, 0.053)***** | | **-0.05 (-0.092, -0.006)*** | **0.022 (0.003, 0.041)*** | |
| Proportion of mediation effect [%] | **-8.4 (-4.3, -1.9)*** | -6.8 (-7.2, .07) | 0.02 (-2.4, 2.0) | | -0.4 (-18.3, 8.0) | 1.0 (-4.0, 8.3) | |

*Significance level at * p< 0.05 ; ** p<0.01, *** p<0.001*
