# Supplementary material for: Reduced cognitive function during a heat wave among residents of non-air-conditioned buildings: An observational study of young adults in the summer of 2016
Source: PLoS Med. 2018 Jul 10;15(7):e1002605. doi: 10.1371/journal.pmed.1002605 (PMC6039003; doi:10.1371/journal.pmed.1002605)
Supplement: S1 Fig — ADD, a 2-digit visual addition/subtraction test; STROOP, the Stroop color-word test. (DOCX) [file pmed.1002605.s005.docx]

S1 Fig. Examples of daily STROOP (S1a, S1b) and ADD (S1c) electronic tests shown in smartphone display


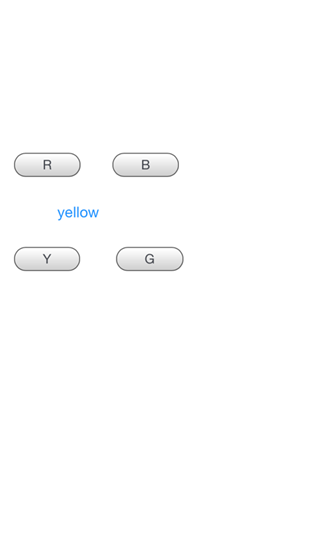

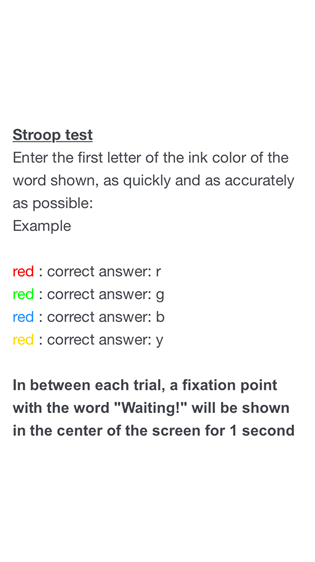


S1 a. Instructions for STROOP Test
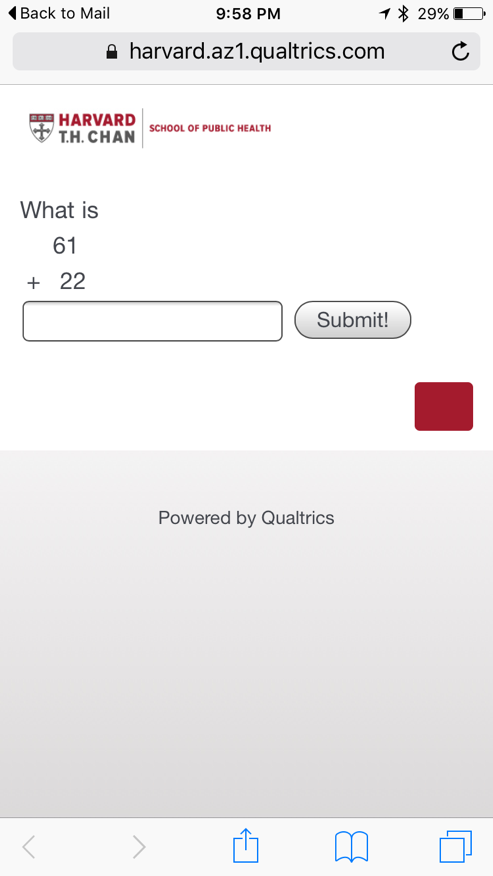
 S1 b. STROOP Test
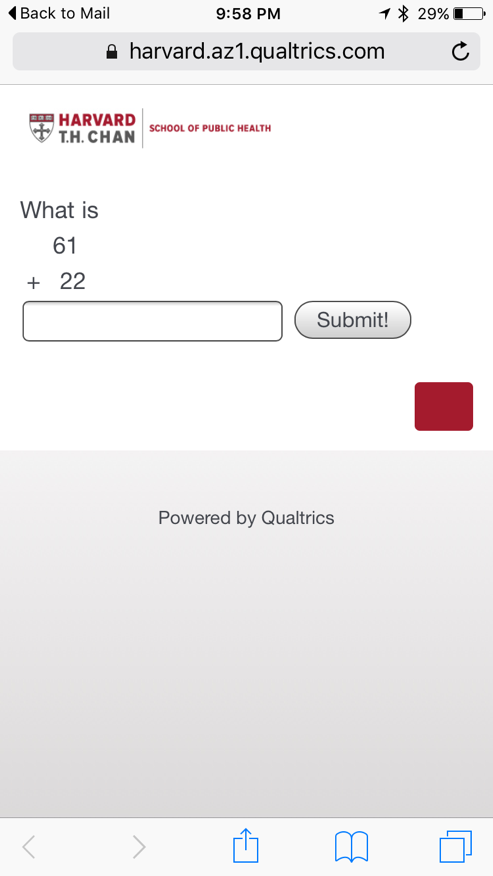
 incongruent trial


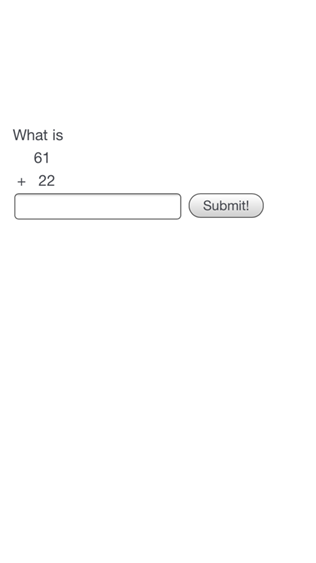


S1 c. ADD Test
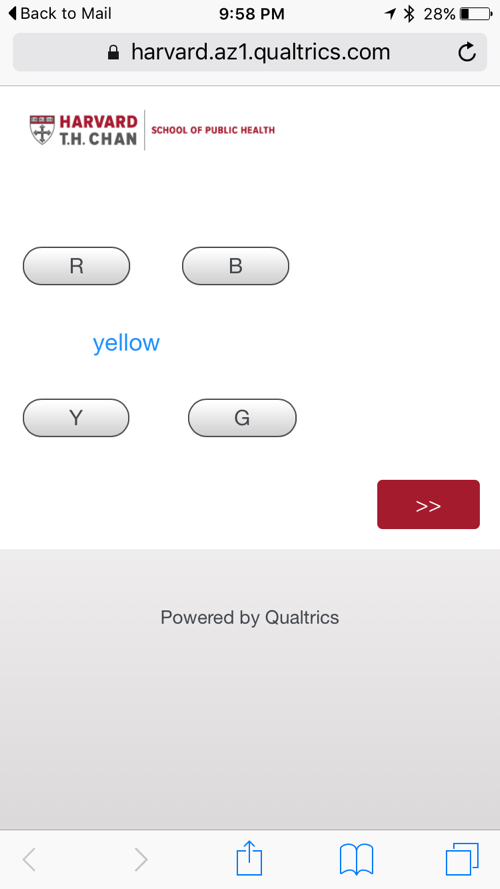

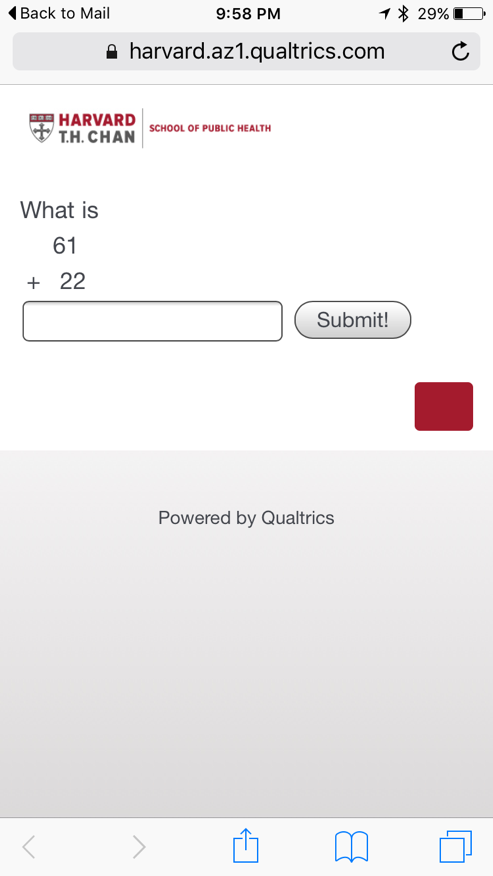
 trial
